# Texas Fever in Native South Carolina Cattle1Read at the Thirty-eighth Annual Meeting of the American Veterinary Medical Association, Atlantic City, N. J., September, 1901.

**Published:** 1902-02

**Authors:** G. E. Nesom

**Affiliations:** College Experiment Station, and State Veterinarian


### TEXAS FEVER IN NATIVE SOUTH CAROLINA CATTLE.[1]

By G. E. Nesom, D.V.M.,
COLLEGE EXPERIMENT STATION, AND STATE VETERINARIAN.

States, like professional men, tend to specialize; but often make sudden and radical changes. The "Palmetto State" has, during its history, specialized in war, indigo, rice, tobacco, nullification, cotton, secession, and the last and most successful is cotton manufactures. However, at no time in its history has South Carolina ever seriously claimed to be a stock-raising State. Possibly there are at present within its borders a score of men who breed high-class Jerseys for sale, half as many who breed Ayrshires, and still less numbers who have small herds of Devons, Holsteins, and Guernseys.

I have been in every county of the State, and have seen only two small herds of Shorthorns, two of Red Polls, and not a single Hereford or Polled Angus. So, while South Carolina cannot be classed as a stock-raising State in the past or at present, it must be said to her credit that many attempts have been made to introduce better cattle, but acclimation fever has promptly claimed most strangers in the herd, and the project has been practically abandoned. It should be remembered in this connection that the whole

[1] Read at the Thirty-eighth Annual Meeting of the American Veterinary Medical Association, Atlantic City, N. J., September, 1901.

of the State from its earliest history has been infested with the common cattle tick; in fact, the history of Texas fever began in South Carolina more than a hundred years ago. For a century it has been produced by cattle from South Carolina[1] when driven into several States further north, and it is likewise known to occur in cattle from Tennessee and the mountainous portions of North Carolina and South Carolina when driven down to middle and lower South Carolina. When John C. Calhoun lived on the historic "Fort Hill" plantation he was very careful not to bring down from the mountains during the summer any cattle purchased there. A friend once wrote him from Tennessee, presenting him with a fine bull, and Mr. Calhoun replied very hastily, thanking him for the remembrance, and also suggested that he keep the bull until winter, when he could be safely sent over without danger of contracting "mountain distemper," a term now known to mean Texas fever. This suggests that in Calhoun's day a large portion of the Piedmont section was free from ticks. Cattle raised there were not immune to Texas fever, and, when exposed to ticks, succumbed to it as readily as they do now. Although the Government quarantine line runs entirely above South Carolina, much of this State is now entirely free from ticks. This result has followed the enactment of a stock law some twenty years ago. Prior to that time the fields were not fenced and stock allowed to run at large. Under this system ticks were widely disseminated every year. Since then stock has been kept up, and many an old citizen has told me that ticks disappeared in certain places soon after the stock law went into effect. The process has gone steadily on ever since, and each year sees many a farm entirely freed from ticks. This is most noticeable in the mountainous portions of the State, and the ticks at present increase in prevalence as we go toward the Atlantic coast. Notwithstanding this general conclusion, there are many farms all over the State which are and have been for several years entirely free from ticks.

Now, since we know that the tick is the common agent in the transmission of Texas fever, we must assume that cattle bred on the tick-free farms of South Carolina are as much subject to this disease as if bred in New Jersey. This may be stating the proposition a little too strong, but it must stand until further investigations are made and other means of transmission more satisfactorily demonstrated.

[1] See Annual Report of the Bureau of Animal Industry for 1899, p. 85.

If stock-feeders buy indiscriminately cattle free from ticks and those infested, the non-immunes will readily pick up a supply of ticks when the entire purchase is pastured and fed together. In the vicinity of cotton-seed oil mills, where native cattle are fed for market, such transactions take place many times during each year, and the loss resulting is considerable.

A few examples from personal observation in my experience as State Veterinarian may serve to illustrate this fact.

In the fall of 1898 a stock-feeder at Winnsboro, just north of Columbia, bought several carloads of native cattle from the surrounding country. One purchase consisted of sixteen head driven down from upper Fairfield and lower Union counties, where ticks are not common. These were put into his pastures and feed-lots along with other purchases carrying ticks. They began dying of Texas fever in fifteen days, and in twenty-five days thirteen of the sixteen were dead.

A son-in-law of this same man lived on a tick-infested farm just outside of Winnsboro. He had lost several head in the same way, and not being an ardent believer in the "tick theory" decided to test its merits. He brought in from tick-free farms two Jersey bulls and put them into his pasture. Both soon became covered with ticks and died in a few days.

A farmer and merchant at Fairplay had extensive Bermuda pastures along the Tugaloo River. In the summer of 1900 he bought in Anderson and Oconee counties, South Carolina, and on the Georgia side of the river, about fifty head of cattle for grazing. Most of those from the South Carolina side were free from ticks, but the Georgia cattle were well sprinkled. As a result the South Carolina cattle contracted Texas fever, and before the summer was over he had lost about one-third of the herd.

The Orr Cotton Mill Company at Anderson fenced about forty acres of slashy land near the mill and gave it as a common pasture for the family cows of the mill hands. These people came in from the surrounding country, some bringing with them tick-infested and some tick-free cattle. In 1900 the pasture became badly infested, and early in September of that year Texas fever appeared. Practically all of the non-immunes died.

A prominent dairyman, whose farm is two and a half miles from the town of Sumter, bought during the winter of 1900–1901 a number of milch cows in Columbia and Sumter. Most of these were raised in town and had never carried ticks. They were placed in his wooded pastures, where ticks are very numerous. About the middle of June, 1901, four or five of these cows died. I was requested to come to the farm at once. On arriving I found that the proprietor was away, and the only symptoms observed by the negroes who attended the cattle were weakness and bloody urine. I proceeded to the pasture and found several more of the newly purchased cattle showing signs of the disease. Rectal temperatures were 104° to 107° F. None of the cattle raised on the farm was affected.

Many other similar examples might be mentioned, but these are sufficient to illustrate the effect on trade in cattle.

Soon after undertaking the work of State veterinarian of South Carolina, in the fall of 1898, I began to receive reports from a few places telling of the losses of cattle from some unknown disease; but no inspections were made until the summer of 1899, when I had a number of reports and requests for inspections. Several trips were made to places along the foot of the mountains, where tick-free and tick-infested farms are almost equally divided, and in each case the deadly malady proved to be Texas fever. But I had much difficulty in convincing the farmers that ticks were responsible for the trouble. The principal arguments used against my diagnosis were statements made in Bulletin No. 26 of the South Carolina Experiment Station, issued in September, 1896, by Dr. W. E. A. Wyman, the former veterinarian of the station. The article referred to constitutes the second part of the bulletin, and is headed "Hæmoglobinuria; Red Water." From it the following extracts are made:

"The various outbreaks of hæmoglobinuria so far investigated by me within the last two years characterize it as a disease attacking the mucous membranes of the stomach, intestines, and urinary organs, with excretion of urine more or less bloody or smoky in color. Invariably I have found that animals taken down with red water had been pastured on low meadows, or there had been low spots in the otherwise more elevated pastures. As a rule, these pastures had a little branch running through them, the banks of which were lined with alder, sumach, bottonwood, oak, and other astringent and irritant plants. These pastures show here and there clumps of shade trees, with pale-looking grass underneath them. So far all outbreaks noted begin eight days to five weeks after the weather in early summer has grown hot and dry. At this time the cattle rest the greater part of the day in the shady spots, browsing on the oak and eating the sickly looking grasses growing underneath the shade trees, actually destroying the foliage and leaving the bare twigs of the various trees and shrubs mentioned above." . . . "The disease is most frequently seen in June and in the fall of the year. Cattle of all ages and conditions are subject to it, while those roughing it all the year round in those pastures are not so readily taken sick, probably from the fact that they are accustomed to the particular food." . . . "Cattle well cared for and suddenly put out on these pastures are more readily attacked." . . . "The food these animals eat (in the pastures) contain some poisonous substance, which is taken up into the blood from the stomach or intestines." . . . "It seems that the peculiar poison taken into the body by the food on entering the blood current breaks down the red blood cells, setting the coloring-matter of these cells free. The blood thus altered reaches the kidneys, which are the great filters of the body, taking out of the blood most of the injurious substances, and in this instance the coloring-matter is filtered out and appears in the urine, giving it the smoky color. The actual nature of the poison concerned in this disease is not yet understood, nor has the Veterinary Department of the South Carolina Experiment Station finished its investigations in regard to this disease."

"Depending on the amount of noxious food eaten, the animal will show more or less symptoms at a variable period." . . . "Some of them, though only slightly diseased, will lie down continually, while the very sick ones divide their time between standing up and resting. All animals immediately give the impression of being very weak, their walk is unsteady, they lose flesh rapidly, and the eye sinks into the head; they no longer chew the cud, they are constipated; pregnant cows slip their calves and seldom recover." . . . "Cows attacked by this disease usually die in three to eight days after they are taken sick." . . . "The muscles have a faint red or yellowish color; the milt, or spleen, is a little larger than usual; kidneys are of ordinary size, showing on section a reddish-brown appearance. The bladder is either filled with dark red urine, or when empty a sediment of a brownish tint is seen." . . . "The gall-bladder is distended with a rather thick bile." . . .

"The first thing that owners usually notice is that the cattle do not come up, or somebody finds a sick or a dead one, and on looking into the trouble others will be found needing attention." . . . "The mucous membrane of the eye or vagina is of a pale or yellowish color." . . . "The pulse beats 70 to 120 per minute." . . . "The temperature runs between 103° to 107° F." . . .

"All pastures known to cause red water are made reasonably safe by either fencing in all shady places or by removing the shade trees in some other way. The same holds good of the shrubs and trees lining the banks of the branches running through these pastures. Hay raised in such meadows is to be fed sparingly, while cattle which have been stall-fed all winter are better given some other food, as millet, barley, green corn, etc, when first put on these pastures."

You can readily see that where a man has thoroughly imbibed these ideas it is hard to induce him to protect his cattle against ticks. At first I tried to collect some statistics regarding the cattle industry of the State and losses from disease, by taking notes where inspections were made; but this method was slow and ineffectual. In the fall of 1900 I acted as cattle inspector of the State Fair, held in Columbia. All tick-free cattle were carefully separated from those carrying ticks. About two hundred head were exhibited, and they were about equally divided between the tick-free and tick-infested. The State Fair Association has a membership of about five hundred, and on returning home I sent to each a copy of the following circular letter:

*Circular of Inquiry Regarding Texas Fever in Cattle.*

OFFICE OF THE VETERINARIAN OF THE SOUTH CAROLINA EXPERIMENT STATION,
CLEMSON AGRICULTURAL COLLEGE.

DEAR SIR: This circular letter is sent you in the hope that you will assist the veterinarian of the Experiment Station in securing some information regarding the cattle disease known as Texas fever.

During the past few years this disease has been prevalent in many sections of the State, but since the passage of the present stock law it has become very common, especially in the up-country in the pastures and pens of stock buyers and feeders.

Texas fever is known by a number of names, but the more important of these are splenic fever, splenetic fever, acclimation fever, Southern cattle fever, tick fever, red water, bloody murrain, bloody urine, distemper, mountain distemper, and many local names.

The symptoms are readily recognized by anyone who has seen cattle suffering from this fever. At first the animal becomes stupid, and leaves the herd for some secluded and shady part of the pasture. They appear listless and droop, as if all energy had forsaken them, the ears drop, the nose is more or less dry, rumination (chewing cud) is suspended, the urine light to dark red in color, and constipation marked. Only small quantities of very dark dung are voided. They are highly fevered, the temperature running from 103° to 107° F. In milch cows the flow of milk is almost suspended. All of the symptoms increase in severity until the animals become almost or quite unconscious, walk around in a circle, and seem to suffer great pain; then convulsions set in, the animal falls, lies on the side, unconscious, and snorting in the intervals between convulsions, until death ensues.

Calves rarely develop the severe symptoms or die from the disease; but in cattle over a year old the death-rate is possibly 50 to 90 per cent., increasing as the age increases.

Post-mortem examination of the carcass shows the flesh to be almost bloodless and pale in color, the spleen (melt) black and easily torn, the bladder filled with bloody urine, the liver and intestines yellowish, and the gall-bladder filled with bile.

In all cases an examination of the skin about the thighs, flanks, neck, and other parts of the body reveals the presence of ticks, which always go with Texas fever. The cause of the disease is a small animal organism (protozoan), which seems at all times to exist in the body of the tick. When the tick inserts its bill through the hide these little germs (?) gain access to the blood of the cow, and there develop, producing a case of Texas fever in ten to twenty days. Death results from the destruction of the red blood cells, the refuse going to the spleen and the coloring-matter to the bladder.

Cattle which have had ticks on them when they were calves are immune to this disease and will not have it again; cattle which have not had ticks on them until a year old will develop the disease as soon as they get the ticks on them.

The Experiment Station officials desire to assist the stockmen of the State in getting this disease under control and preventing serious losses in future. Inoculation experiments are now in progress, and it is hoped that immunity to it may be produced by artificial means.

You are requested to answer the questions on the inclosed postal card and return as soon as possible to the Veterinarian, who wishes to thank you in advance for your co-operation in the matter.

Yours truly,

G. E. NESOM,

Veterinarian.

The postal card sent was as follows:

NOTICE: Please answer the following questions:

Name, P. O., S. C.
How many head of cattle have you on hand?
How many of these were raised on your farm?
Number bought in 1900 Number sold in 1900
Did your cattle have ticks on them in 1900?
If not, how long since they have had?
Have any of your cattle had Texas fever?
If so, how many? How many died?
Are ticks common on the cattle of your section?
Give names and addresses of the largest stock-owners in your section.

Only about 10 per cent. of these cards were returned filled out, but they came from twenty-four of the forty counties, and represented every section of the State. From them the following statistics are taken:

| Counties. | No. reports. | No. reported. | Tick free. | Infected. |
|---|---|---|---|---|
| Abbeville | 1 | 26 | 00 | 26 |
| Anderson | 2 | 123 | 123 | |
| Beaufort | 1 | 14 | 14 | |
| Barnwell | 1 | 13 | 13 | |
| Charleston | 2 | 67 | 17 | 50 |
| Chester | 5 | 138 | 138 | |
| Darlington | 4 | 124 | 90 | 30 |
| Edgefield | 1 | 19 | 19 | |
| Fairfield | 4 | 180 | 97 | 83 |
| Florence | 1 | 25 | 25 | |
| Greenville | 3 | 14 | 14 | |
| Greenwood | 4 | 118 | 118 | |
| Hampton | 2 | 13 | 13 | |
| Kershaw | 1 | 12 | 12 | |
| Laurens | 3 | 58 | 16 | 42 |
| Marion | 3 | 208 | 18 | 190 |
| Marlboro | 3 | 22 | 20 | 2 |
| Newberry | 3 | 32 | 21 | 11 |
| Oconee | 4 | 89 | 89 | |
| Orangeburg | 2 | 29 | 29 | |
| Pickens | 2 | 45 | 45 | |
| Richland | 13 | 554 | 175 | 379 |
| Spartanburg | 1 | 3 | 3 | |
| York | 4 | 151 | 66 | 85 |
| Totals | 70 | 2077 | 1175 | 898 |

From the above figures you see that these men report 55.6 per cent. of their cattle free from ticks, while 34 per cent. are infested. The number of deaths in 1900 from Texas fever among these 2077

head was 55, or 2.5 per cent. From personal observation I am sure this is quite a low estimate. The number and value of cattle in South Carolina[1] for the year 1898 was:

| | |
|---|---|
| 126,762 milch cows, valued at $16.75 each . | $2,123,264 |
| 141,509 other cattle, valued at $9.45 each . | 1,337,399 |
| Total value of all cattle . . . . | $3,460,663 |
| Annual loss from Texas fever, 2.65 per cent. . | 91,767 |

I have publicly stated on several occasions that I believe the annual loss from this disease in South Carolina is $10,000. I am now fully convinced that I could have made it $50,000 with perfect safety; but the actual loss is not reducible to figures, and the general conclusion is that the stock-owners of South Carolina have a very important problem to handle. They are "between the devil and the deep blue sea"—neither with nor without cattle ticks. The breeder in the upper country cannot sell his cattle to men in the low country, nor can he bring the tick-infested cattle of the low country to his tick-free farm. On the other hand, the low country man loses practically all cattle purchased from the mountainous portions. Either transaction causes both immediate financial loss and injury to the business interests of breeders. The result is that the cattle business is in a measure paralyzed. The number and value of cattle decreases every year. At the same time our cities and towns are rapidly becoming manufacturing centres, and the farms are being drained of white labor, which goes to the cotton mills, where they cease to be producers of food; consequently there is an ever-increasing demand for milk, butter, and beef. How are these demands to be met?

The answer must come from intelligence, energy, and business enterprise. The State must be separated into two divisions, the upper portion entirely freed from ticks and the lower portion rigidly quarantined. Better blood must be introduced, and inoculation persistently practised on foundation stock for the permanently infested section. Those farms in the low country, which must by virtue of environment be surrounded by tick-infested farms for many years, although they are now free from ticks, should be at once reinfested, in order that the cattle bred there may not be subject to Texas fever. This is not a step backward, but a necessary precaution.

The abandoned cotton fields must be converted into verdant

[1] See Annual Report of the Bureau of Animal Industry for 1899.

pastures, meadows stocked with the choicest grasses adapted to this section, and modern dairying methods introduced; then the lowing herd will bring peace, plenty, and happiness to the home of the Palmetto farmer.

